# RiboA: a web application to identify ribosome A-site locations in ribosome profiling data

**DOI:** 10.1186/s12859-021-04068-w

**Published:** 2021-03-25

**Authors:** Danying Shao, Nabeel Ahmed, Nishant Soni, Edward P. O’Brien

**Affiliations:** 1 Institute for Computational and Data Sciences, Pennsylvania State University, University Park, USA; 2 Department of Chemistry, Pennsylvania State University, University Park, USA

**Keywords:** Ribosome profiling, Web application

## Abstract

**Background:**

Translation is a fundamental process in gene expression. Ribosome profiling is a method that enables the study of transcriptome-wide translation. A fundamental, technical challenge in analyzing Ribo-Seq data is identifying the A-site location on ribosome-protected mRNA fragments. Identification of the A-site is essential as it is at this location on the ribosome where a codon is translated into an amino acid. Incorrect assignment of a read to the A-site can lead to lower signal-to-noise ratio and loss of correlations necessary to understand the molecular factors influencing translation. Therefore, an easy-to-use and accurate analysis tool is needed to accurately identify the A-site locations.

**Results:**

We present RiboA, a web application that identifies the most accurate A-site location on a ribosome-protected mRNA fragment and generates the A-site read density profiles. It uses an Integer Programming method that reflects the biological fact that the A-site of actively translating ribosomes is generally located between the second codon and stop codon of a transcript, and utilizes a wide range of mRNA fragment sizes in and around the coding sequence (CDS). The web application is containerized with Docker, and it can be easily ported across platforms.

**Conclusions:**

The Integer Programming method that RiboA utilizes is the most accurate in identifying the A-site on Ribo-Seq mRNA fragments compared to other methods. RiboA makes it easier for the community to use this method via a user-friendly and portable web application. In addition, RiboA supports reproducible analyses by tracking all the input datasets and parameters, and it provides enhanced visualization to facilitate scientific exploration. RiboA is available as a web service at https://a-site.vmhost.psu.edu/. The code is publicly available at https://github.com/obrien-lab/aip_web_docker under the MIT license.

## Background

Regulation of the translation process influences steady-state protein levels in cells. Hence, it is important to understand translation to determine its role in gene expression. The development of ribosome profiling (Ribo-Seq), a high-throughput Next-Generation Sequencing (NGS) method, has greatly advanced the transcriptome-wide study of translation [1–3]. The A-site on a ribosome-protected mRNA fragment is the codon that was being translated by the ribosome at the time translation was halted in a Ribo-Seq experiment. The exact A-site location may not be critical in some of the global gene-level analysis of Ribo-Seq data, for example, the total number of reads is more important in Ribo-Seq analysis studying differential mRNA expression. However, it is essential to accurately identity the A-site when studying fine-grained aspects of translation, such as codon translation rates. Misassignment of A-site can lead to lower signal-to-noise ratio and loss of correlations that indicate a biological effect. Therefore, an easy-to-use analysis tool that can accurately identify A-site locations is needed.

The A-site location is identified by an offset value, which is the number of nucleotides separating the start of the A-site codon from the 5′-end of the ribosome-protected mRNA fragment. For example, in one study [1], the A-site location has been estimated to be 15 nucleotides from the 5′-end of ribosome-protected mRNA fragments that are 28 nucleotides in length. In the past, a constant heuristic offset has been used for a wide range of fragment sizes. A constant offset of 15 nt has been applied to Ribo-Seq data from *S. cerevisiae* [4–6] and mouse embryonic stem cells (mESCs) [7, 8]. This approach neglects potential variations in the offset value as a function of fragment length and reading frame the 5′-end nucleotide is in. Such variation could arise from incomplete digestion of mRNA or stochastic mRNA cleavage that can happen at either end of an mRNA fragment during a Ribo-Seq experiment. Both events lead to fragments with different sizes and potentially different A-site offsets. It is even possible that fragments of the same length have different offset values. Therefore, a constant offset for all fragment sizes is insufficient to describe the A-site location. A number of software tools have been developed to identify offset values using sophisticated algorithms, such as Python packages Plastid [9] and scikit-ribo [10], and R packages RiboProfiling [11], riboWaltz [12] and RiboVIEW [13].

Recently, a novel method was created that utilizes Integer Programming [14]. This method embodies the fact that the A-site of actively translating ribosomes must always be located between the second codon and the stop codon of a transcript [15], and utilizes all the mapped reads in and around the coding sequence (CDS). This constraint turns A-site identification into an optimization problem. In an earlier study it was shown to generate the most accurate A-site offset values to date [14]. However, this method has not been packaged for easy use by the community. Further, the original source code was written in deprecated Python 2 and leveraged external software, which presents a barrier to using the method.

Therefore, we developed RiboA, a user-friendly web application that employs this Integer Programming method. RiboA identifies the most accurate A-site location on a ribosome-protected mRNA fragment and generates the A-site read density profiles. In addition, RiboA tracks all the input datasets and parameters, and hence supports reproducible analyses. It also provides enhanced visualization to facilitate scientific exploration. RiboA is containerized with Docker [16], and it can be easily ported across platforms.

## Implementation

The homepage of RiboA introduces the Integer Programming method for identifying A-site locations and includes a tutorial and a video on how to use RiboA. While login is not required and anonymous users can submit jobs, they are limited to using the existing input datasets hosted on the server. These include a published dataset for *S. cerevisiae* [17], a pooled dataset for *E. coli* [18–20], and a dataset for mESCs [21]. On the other hand, users who have logged in are able to utilize more features, such as uploading their own datasets and reviewing their job histories. To login, users can either use their existing Google account or sign up with RiboA. Users can also choose to download the package, set up their own application and mount their dataset repository by following the instructions on our GitHub repository.

**Fig. 1:**
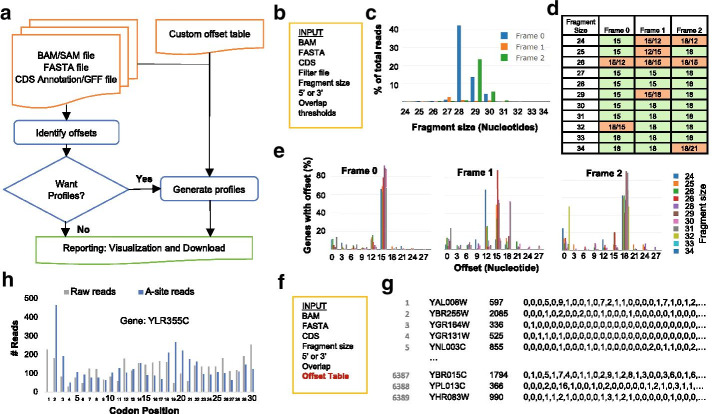
Overview of RiboA. **a** Workflow diagram of using RiboA; **b** input datasets and parameters for an offset job; **c**–**e** results generated from an offset job: **c** distribution of mRNA fragment size for Frame 0 (blue), Frame 1 (orange) and Frame 2 (green) respectively, **d** A-site offset table where green indicates that the offset value can be uniquely identified for the fragment size and frame combination while orange indicates that a unique offset cannot be identified and the top two probable offsets are shown in such scenarios, and **e** distribution of A-site offset among genes; **f** input datasets and parameters for a profile job; **g** A-site read density profiles generated from a profile job where each line represents one gene and lists the gene name, number of nucleotides for the gene and read counts for each nucleotide after applying the A-site offset table; **h** comparison between the raw reads mapped by 5′-end (grey) and the reads after applying the A-site offset value (blue) for the first 30 codons in gene YLR355C. **c** and **e** are generated directly from the RiboA web application, and **h** is plotted in Microsoft Excel by using the data generated from the RiboA web application

The workflow diagram of using RiboA is illustrated in Fig. 1a. There are two types of jobs: an offset job and a profile job. The purpose of an offset job is to determine the A-site offsets that can then be used to determine the A-site profiles if needed. RiboA calculates the A-site offset, denoted *∆*, for fragments of a particular size (*S*) and frame (*F*) that map onto gene *i* by maximizing the total number of reads between the second codon and stop codon of a transcript *T*(*∆*│*i, S, F*), subject to constraints 0 ≤ *∆* ≤ *S* and *∆* mod 3 = 0. Sometimes the top two highest scores *T*(*∆′*│*i, S, F*) and *T*(*∆″*│*i, S, F*) can be very close, where *∆′* and *∆″* are the two corresponding offset values. To avoid bias caused by small sample size, genes who on average have less than one read per codon are filtered out. To further improve the robustness of the offset table, we implemented two additional thresholds to identify unique offsets. First, at least 70% of genes should exhibit the most probable offset and at least ten genes should be present in each dataset. This threshold can be lowered to a value above 50% to generate more uniquely identified offsets. However, a lower threshold may lead to less accurate results and it is up to the researcher’s discretion. Secondly, the average number of reads in the second, third and fourth codon is at least five times the number of reads in the first codon. Three of the thresholds can be customized when the user submits a RiboA offset job (see below). Note that if the A-site location cannot be uniquely determined the top two offset values will be included in the generated offset table, and we recommend users disregard reads with uncertain A-site offsets.

On the offset job submission page, users need to provide the input datasets and parameters (Fig. 1b). The input datasets include (1) a BAM or SAM file containing the raw sequence alignments of Ribo-Seq reads, (2) a FASTA file containing the sequences of the transcripts, and (3) a GFF or CDS annotation file. Note that the GFF option is only available for *S. cerevisiae* and *E. coli.* If the user chooses to upload a GFF file, RiboA will convert it to the CDS annotation file in the backend. The format of the CDS annotation file is specific to the alignment mode, *e.g.* genome or transcriptome. Upon a user’s selection of the alignment mode, the corresponding instruction for the annotation format is shown on the webpage. We have provided examples for both the GFF files and the TAB-based annotation files on the “Upload Data” page of the website. A number of parameters can be customized, such as the range of fragment size measured in nucleotides and the number of nucleotides beyond the CDS region of a gene which are to be avoided to overlap with another gene, the minimum average number of reads per codon for filtering genes, the minimum percentage of genes with the most probable offset for assigning a unique offset, and the minimum ratio between the average reads in the second, third and fourth codon and the reads in the first codon also for assigning a unique offset. These parameters help to improve the robustness of the method. Optionally, users can also upload a filter file to include or exclude genes from the analysis.

A profile job takes in a custom offset table for specified read lengths and reading frames and generates A-site read density profiles mapped to genes. A default offset table is provided for *S. cerevisiae* that was used in our previous study [14]. Other input dataset and parameters to a profile job are similar to an offset job (Fig. 1f).

Depending on the input file size, the job may take over one hour to run for a 3 GB SAM file with 5 million alignments. Thus, we utilize a redis message broker (https://redis.io) and a celery (https://docs.celeryproject.org) task queue to orchestrate jobs asynchronously. Upon job submission, users are immediately redirected to the job’s reporting page that will show that the job is in the status PENDING or RUNNING; while in the background, the job is appended to a queue and multiple workers take on jobs from the queue sequentially. Once the job finishes, an email notification with the link to the reporting page is sent to the user. If the job finishes successfully, the reporting page provides the output files to download and visualizes some of the important results. The input datasets and parameters are also tracked and shown on the reporting page so that the analysis can be easily reproduced. If the job fails, users can review the log file for causes.

The web application is containerized with Docker. It consists of five services: (1) a Django (https://www.djangoproject.com) web service, (2) a nginx (https://www.nginx.com) web server, (3) a redis message broker, (4) a celery asynchronous job queue, and (5) a PostgreSQL (https://www.postgresql.org) database. Each service resides in its own Docker container and the five containers are connected with Docker Compose. Containerization makes it easy to port the application across platforms and deploy it to a cloud. In addition, users can simply set up a local environment and run it as a standalone application on their own machines.

The front-end of the web application utilizes multiple JavaScript and CSS libraries to improve user experience. For example, Plotly generates interactive visualizations and Bootstrap renders responsive mobile-friendly webpage styles.

## Results and discussion

A RiboA offset job generates the A-site offset table (Fig. 1d) along with the supporting results, including fragment size distribution (Fig. 1c), offset distribution among genes (Fig. 1e), and the number of genes for various fragment size and frame combinations. The Ribo-Seq dataset used in Fig. 1 is an *S. cerevisiae* dataset published in Jan et al*.* [22] and the parameters were set to the default values. The offset table is color coded where green indicates that the most probable offset value can be uniquely identified for that fragment size and frame combination, while orange indicates that the offset value cannot be uniquely identified and both of the top two most probable offset values are listed. The offset table in Fig. 1d shows that although the most probable offset value is often either 15 nt or 18 nt, it does vary between fragment sizes and frames. Figure 1c shows that the fragment size spans a wide range, possibly due to incomplete digestion of RNA and stochastic mRNA cleavage as mentioned before. Figure 1e gives a more granular view into the offset distribution and validates the offset table in that most genes have an offset of 15 nt or 18 nt. Both Fig. 1c and Fig. 1e are rendered interactively on RiboA’s reporting page. For example, the corresponding numbers will show up when the lines are hovered on, and the figures can be zoomed in and out. In Fig. 1e, users can temporarily exclude a fragment size by clicking off the corresponding legend. With a less crowded figure, users can focus on the range of fragment sizes that they are interested in. In sum, both figures assist in verifying the quality of the input datasets and the validity of the resulted offset table, and the interactive visualization presented by RiboA facilitates the data exploration.

RiboA outputs three sets of A-site density profiles: (1) the A-site reads per nucleotide, (2) the A-site reads per nucleotide mapped to Frame 0 by applying the transformation that for reads in frame 1 and 2 the offset is reduced by 1 and 2, respectively, (3) the A-site reads per codon. Each A-site read density profiles generated from RiboA is contained in a tab file (Fig. 1g). In the file, each line represents one gene, starting with the gene name and the number of nucleotides (or codon) for the gene, followed by a list of read counts for each nucleotide (or codon) after applying the A-site offset table. In Fig. 1h, we also compared the raw reads mapped by 5′-end (grey) with the mapped reads after applying the A-site offset table (blue). Figure 1h shows the first 30 codons comparison for gene YLR355C. Note that the blue line has a spike at the second codon, which is expected because of the time taken by the ribosome to initiate translation with the start codon in the P-site. With the generated A-site density profile, users can further create meta-gene analysis around the start and stop codons to ensure that there are reasonable densities for this method to be applied.

**Fig. 2:**
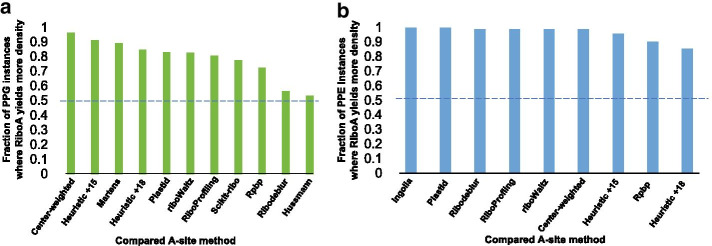
Compare RiboA with other A-site methods. **a** The fraction of PPG instances where RiboA yields higher ribosome density at glycine than other methods; **b** The fraction of PPE instances where RiboA yields higher ribosome density at glutamatic acid than other methods. The figures are generated in Microsoft Excel

To verify that RiboA generates the most accurate A-site offsets, we examined the ribosome density at the strongest stalling PPX motifs assigned by RiboA in comparison with other A-site methods. To date, this is the best approach to compare A-site methods because these stalling motifs have been identified by both Ribo-Seq and biochemical studies, and the A-site location is known to be at the codon encoding the third residue of the motif [23–25]. We first compared the ribosome density at the stalling motif PPG in *S. cerevisiae* using a pooled dataset [14] (Fig. 2a). The A-site methods we compared with RiboA include the heuristic 15 nt offset described before, a heuristic 18 nt offset, and offsets generated by a number of other methods including center-weighting [26], the Hussmann method [27], the Martens method [28], Rpbp [29], plastid [9], RiboProfiling [11], Ribodeblur [30], Scikit-ribo [10], and riboWaltz [12]. We found that RiboA yields significantly higher ribosome density at glycine than almost all the other methods (Wilcoxon signed-rank test *P* < 0.05 with n = 224). The only exception is the Hussmann method where the difference is not statistically significant.

We then examined the stalling motif PPE in an mESCs dataset [21] (Fig. 2b). We didn’t include the Hussmann method [27], the Martens method [28] and Scikit-ribo [10] due to the limitations of these tools when applied to mESCs datasets. We added the method presented in Ingolia et al*.* [21] where incremental offset values were assigned to stratified fragment sizes. Here, RiboA yields significantly higher ribosome density than all the other methods (*P* < 0.05 with n = 104). Therefore, RiboA is the most accurate tool in identifying A-site locations.

We note that the heuristic constant offsets, *e.g.* 15 nt and 18 nt, perform relatively well in some of the cases, and have previously proven useful in studying translation properties. However, RiboA gives more accurate offset in most cases, and hence better signal-to-noise ratio in Ribo-Seq analysis.

RiboA assumes the ribosomes are undergoing steady-state translation, and it can only be applied to steady-state ribosome profiling data. RiboA is not appropriate for datasets from non-steady-state experiments, such as the ribosome run-off experiments where initiation is blocked by antibiotics, such as harringtonine treatment.

## Conclusions

RiboA is a web application that identifies A-site locations and generates read density profiles. We have shown that RiboA is the most accurate in identifying A-site on Ribo-Seq mRNA fragments compared to other tools. In addition, by monitoring all the input datasets and parameters, RiboA supports reproducible computation. And the interactive visualization it presents can facilitate the scientific exploration. As a user-friendly web application, the use of RiboA requires zero programming skills. The containerization has further increased its portability. Detailed tutorials on how to use RiboA and on how to setup the container have been provided both on the RiboA’s home page and the GitHub repository. By making this tool easier to use, we hope RiboA will find widespread use by the community.

### Availability and requirements

*Project name*: RiboA.

*Project home page*: A-site.vmhost.psu.edu.

*Operating system(s)*: Platform independent.

*Programming language*: Python, PostgreSQL, HTML, JavaScript, CSS.

*Other requirements*: Web browser.

*License*: MIT license.

*Any restrictions to use by non-academics*: None.

## Data Availability

RiboA is available as a web service at https://a-site.vmhost.psu.edu/. The code is publicly available at https://github.com/obrien-lab/aip_web_docker under the MIT license.

## Abbreviations

CDS: Coding sequence
NGS: Next-generation sequencing
mESC: Mouse embryonic stem cell
Ribo-Seq: Ribosome profiling
